# Detection of fowl adenovirus D strains in wild birds in Poland by Loop-Mediated Isothermal Amplification (LAMP)

**DOI:** 10.1186/s12917-020-2271-4

**Published:** 2020-02-14

**Authors:** Jowita Samanta Niczyporuk, Wojciech Kozdruń, Hanna Czekaj, Natalia Styś-Fijoł, Karolina Piekarska

**Affiliations:** Department of Poultry Diseases, National Veterinary Research Institute, Partyzantow 57, 24-100 Pulawy, Poland

**Keywords:** Interspecies transmission, LAMP, Virus strains, Wild birds

## Abstract

**Background:**

The present study on the role of strains of adenovirus in wildlife reservoirs, and their prevalence is under exploration. In several previous studies, the presence of adenovirus strains in wild birds has been investigated. Worldwide distribution and outbreaks of adenovirus infections have been reported by many authors. The present study investigated the prevalence of FAdVs in 317 samples of different bird species from the northwestern region of Poland. An applied specific, sensitive, and efficient, without cross-reactivity loop-mediated isothermal amplification (LAMP) method to gauge the prevalence of fowl adenovirus strains in wild birds was developed and used.

**Results:**

The method was based on the sequence of the loop L1 HVR1–4 region of the hexon gene of the FAdV genome reference strains FAdV-2 KT862805 (ANJ02325), FAdV-3 KT862807 (ANJ02399) and FAdV-11 KC750784 (AGK29904). The results obtained by LAMP were confirmed by real-time PCR. Among 317 samples obtained from wild birds, eight FAdV isolates (2.52%) were identified and produced a cytopathic effect (CPE) in chicken embryo kidney cells (CEK). Three FAdV types belonging to species Fowl adenovirus D were detected, which were isolated from three adenovirus types 2/3/11, and have been confirmed in three mute swans (*Cygnus olor*), three wild ducks (*Anas platyrhynchos*), one owl *(Strigiformes),* and one common wood pigeon (*Columba palumbus*).

**Conclusions:**

This study provides the first accurate quantitative data for the replication of fowl adenovirus strains in wild birds in Poland, indicating adenovirus interspecies transmission, and demonstrating the circulation of FAdVs in wild birds.

## Background

Adenoviruses belong to the *Adenoviridae* family, and are non-enveloped double-stranded DNA viruses [1]. The conserved domains are responsible for specific structure of molecule, and for trimer formation [2, 3]. The highly variable domains are located mainly outside of the virion, and are responsible for the antigenic properties of the virus strains [1, 4]. The International Committee on the Taxonomy of Viruses [2] separated the *Adenoviridae* family into five genera: *Mastadenovirus, Aviadenovirus, Siadenovirus, Atadenovirus,* and *Ichtadenovirus*. Fowl adenoviruses (FAdV) are separated into five species designated as *Fowl adenovirus A* to *Fowl adenovirus E* with 12 types *Fowl adenovirus 1-8a-8b* and *11*. FAdVs are a very divergent pathogens with generally a low level of virulence [4], however, under certain conditions, they can cause a variety disorders in domestic and wild birds [2, 4, 5]. Adenoviral infections may manifest themselves as asymptomatic or as complication factors in the course of different diseases [1, 4]. They can be infectious for fish, reptiles, amphibians, birds, and mammals, and have been isolated from over 40 vertebrate species [1, 2, 6, 7]. Wild birds with fowl adenovirus infections have been documented by Kumar [8], being presented in falcons [9–11], common buzzards [12], black kite [8], tawny frogmouths [13], psittacines [7, 14, 15] and pigeons [16]. It is possible that under some conditions, fowl adenoviruses may be more virulent in non-host-adapted species than in their typical ones [4]. Some virulent strains, can pass the species barrier, and infect new organisms [4, 17]. They can transmit horizontally [4], and vertically [18].

The detection of fowl adenoviruses is very important from an epidemiological point of view. Gunes [19] developed real-time PCR for the detection and quantitation of FAdV(A-E) species, however, in their study, the designed primers were based on the conserved nucleotide sequence of the 52 K gene with an efficiency of 98%, and regression square values of *R*^2^ = 0.999. Different real-time PCR and a subsequent high-resolution melting curve analysis (HRM) of the 191 bp region of the hexon gene, and restriction enzyme analysis have been performed by Steer et al. [20] for differentiating all FAdVs species, and the melting curve profiles were found to be related mainly to GC composition and distribution through the amplicons.

The objective of the present study was to estimate the number of wild birds infected by FAdVs in a forest environment, estimated the geographical distribution of this agent in the region, and perform and applied loop-mediated isothermal amplification for the detection of fowl adenovirus strains and designation the adenovirus species/types.

## Results

### Sample collection

The specification of wild bird species which have been used for the study were presented in Table 1.

**Table 1:** Specification of wild bird species used for the study

Species	Number of birds	Places	Region
Mallard (*Anas platyrhynchos*)	9/3^a^	Kuźnia	Pomeranian voivodeship
Crow (*Corvus corone* cornix)	40	Kuźnia	Pomeranian voivodeship
Garganey (*Anas querquedula*)	13	Gdynia	Pomeranian voivodeship
Common Cuckoo (*Cuculus canorus*)	4	Gdańsk	Pomeranian voivodeship
Pied Flycatcher (*Ficedula hypoleuca)*	3	Reda	Pomeranian voivodeship
Common Kestrel (*Falco tinnunculus*)	3	Stara Kiszewa	Pomeranian voivodeship
Jackdaw (*Corvus monedula)*	13	Gdańsk	Pomeranian voivodeship
Song Thrush (*Turdus philomelos*)	12	Kartuzy	Pomeranian voivodeship
Magpie (*Pica pica)*	10	Gdynia	Pomeranian voivodeship
Whitethroat (*Sylvia communis)*	1	Starkowo	Pomeranian voivodeship
Willow Warbler (*Phylloscopus trochilus)*	4	Kiezmark	Pomeranian voivodeship
Thrush Nightingale (*Luscinia luscinia)*	7	Władysławowo	Pomeranian voivodeship
Common Guillemot (*Uria aalge*)	6	Trąbki Wielkie	Pomeranian voivodeship
Velvet Scoter (*Melanitta fusca)*	4	Nowy Dwór	Pomeranian voivodeship
Black-headed Gull (*Chroicocephalus ridibundus*)	2	Osówek	Pomeranian voivodeship
Great Crested Grebe *(Podiceps cristatus)*	1	Żukowo	Pomeranian voivodeship
Robin (*Erithacus rubecula*)	9	Pruszcz Gdański	Pomeranian voivodeship
Long-tailed Tit *Aegithalos caudatus*	2	Piła	Pomeranian voivodeship
Montagu’s Harrier (*Circus pygargus*)	1	Cedry Wielkie	Pomeranian voivodeship
Common wood pigeon (*Columba livia*)	1^a^	Dobrogoszek	Pomeranian voivodeship
Goldcrest *(Regulus regulus)*	5	Kościerzyna	Pomeranian voivodeship
Common Treecreeper *(Certhia familiaris)*	2	Reda	Pomeranian voivodeship
Blackcap (*Sylvia atricapilla*)	2	Borzestowska huta	Pomeranian voivodeship
Mute swans (*Cygnus olor*)	4/3^a^	Luzino	Pomeranian voivodeship
Common buzzard (*Buteo buteo)*	2	Sierakowice	Pomeranian voivodeship
Raven (*Corvus corax)*	5	Lębork	Pomeranian voivodeship
Goshawk (*Accipiter gentilis)*	17	Nowy Dwór	Pomeranian voivodeship
Common pheasant (*Phasianus colchicus*)	14	Tczew	Pomeranian voivodeship
Common Chiffchaff (*Phylloscopus collybita)*	1	Rusociń	Pomorskie voivodeship
Common Swift (*Apus apus*)	9	Miechucino	Pomorskie voivodeship
Capercaillie (*Tetrao urogallus*)	1	Reda	Pomorskie voivodeship
Eurasian Coot (*Fulica atra*)	11	Sopot	Pomeranian voivodeship
White Stork (*Ciconia ciconia*)	27	Goręczyno	Pomeranian voivodeship
Spotted Flycatcher *(Muscicapa striata*)	3	Dobrzewino	Pomeranian voivodeship
Paridae *(Passeriformes)*	11	Kowalewo	Pomeranian voivodeship
Long-tailed Duck (*Clangula hyemalis*)	8	Chojnice	Pomeranian voivodeship
Great Cormorant (*Phalacrocorax carbo)*	4	Strzebielino	Pomeranian voivodeship
Horned Grebe *(Podiceps auritus*)	2	Lewinko	Pomorskie voivodeship
Common Merganser (*Mergus merganser*)	1	Chojnice	Pomorskie voivodeship
Grey Partridge (*Perdix perdix)*	2	Gdańsk	Pomorskie voivodeship
Eurasian Bittern *(Botaurus stellaris*)	5	Bojano	Pomorskie voivodeship
Common Blackbird *(Turdus merula)*	1	Gdynia	Pomorskie voivodeship
Great Tit (*Parus major*)	1	Gdańsk	Pomeranian voivodeship
Owl *(Strigiformes)*	1/1^a^	Goręczyno	Pomeranian voivodeship
Eurasian Siskin (*Carduelis spinus*)	1	Czapielska	Pomeranian voivodeship
Eurasian Reed Warble *(Acrocephalus scirpaceus)*	2	Jagatowo	Pomeranian voivodeship
Lesser Whitethroat *(Sylvia curruca)*	1	Przodkowo	Pomeranian voivodeship
Garden Warbler *Sylvia borin*	13	Tuszkowy	Pomorskie voivodeship
Common Cuckoo (*Cuculus canorus*)	17	Sobieszewo	Pomeranian voivodeship
			Total
			317

^a^positive birds for adenovirus infection

### Virus isolation

**Fig. 1:**
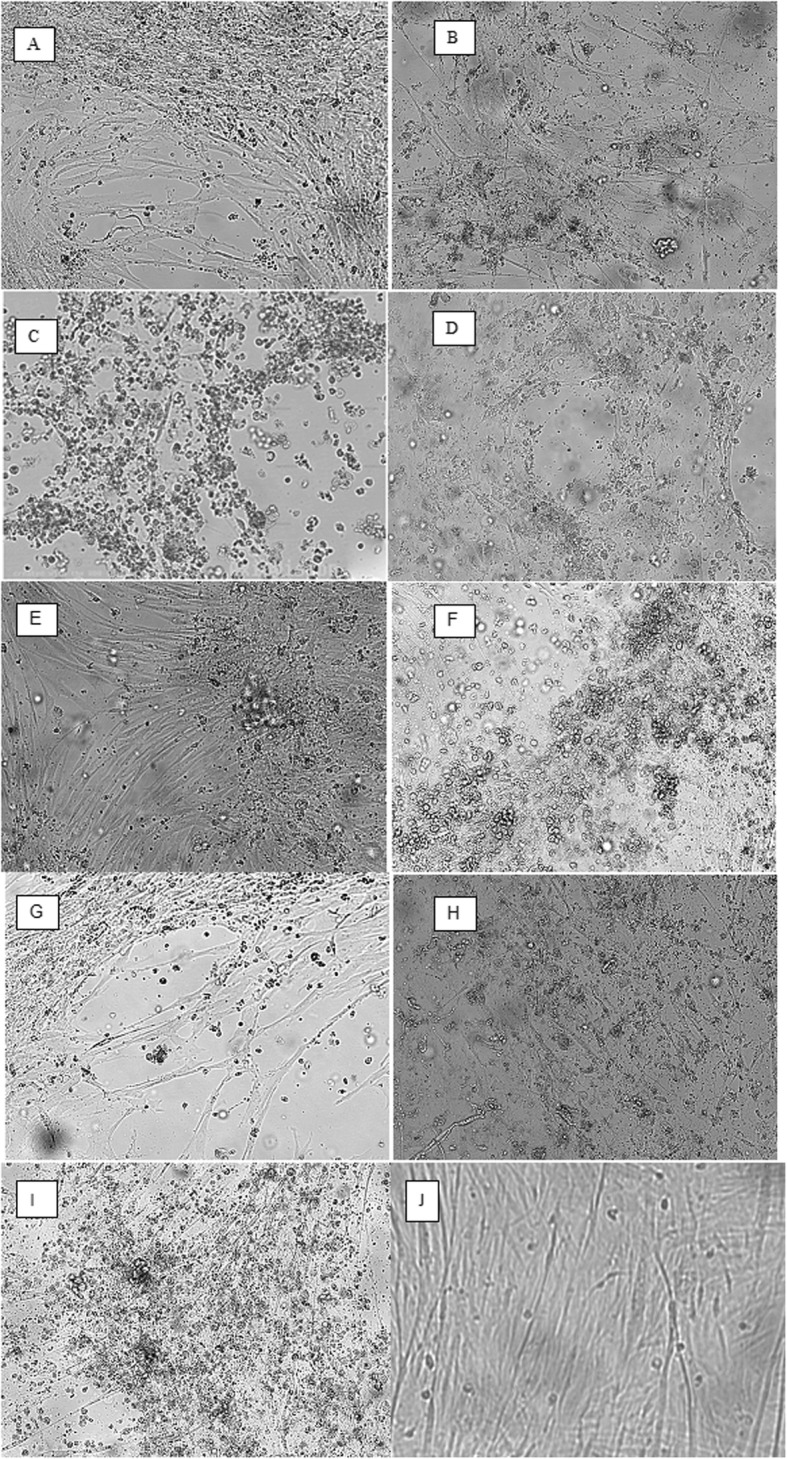
**a**-**h** Characterisation of growth of FAdV strains in CEK cells. Observation on formation of CPE Characteristic cytopathic effect were observed at 96 h after inoculation with the 3rd passage of FAdVs strains. The TCID_50_ of the strains were between 10^4.0^/ml to 10^5.5^/ml on CEK cultures **a** -1/Msw1/16–2/D, **b** -2/Msw2/16–2/D, **c** -3Msw3/16–2/D, **d** -4Wd1/16–2/D, **e** 5-Wd2/16–11/D, **f** 6-Wd3/16–11/D, **g** 7-owl1/16–11/D, **h** 8-wpi1/16–11/D. **i**-**j** Positive control, CEK cultures infected with adenovirus strain FAdV-2/D in doses of 10^3.0^TCID_50_, negative control, non infected CEK cultures. The pictures were taken at 72 h after inoculation

The field and reference strains were propagated in CEK cultures. Three passages were conducted, each of them for 96 h. In the third passage, the first CPE was recognised in about 18–24 h post infection. The cells were bigger, rounder, and filled with granules. Over the following days, the quantity of damaged cells increased and covered the surface of the bottles. Changes in the pH of the medium were observed, and these also had an influence on the damaged cells. CPE with different intensity was observed and presented in Fig. 1a-j field strains A)-1/Msw1/16 (Msw = mute swan), B)-2/Msw2/16 (Msw = mute swan), C)-3Msw3/16 (Msw = mute swan), D)-4Wd1/16 (Wd = wild duck), E)5-Wd2/16 (Wd = wild duck), F)6-Wd3/16 (Wd = wild duck), G)7-owl1/16 (owl = owl)*,* H)8-wpi1/16 (wpi = wood pigeon). Positive and negative controls are also presented in Fig. 1a-j.

### Virus identification and detection

**Fig. 2:**
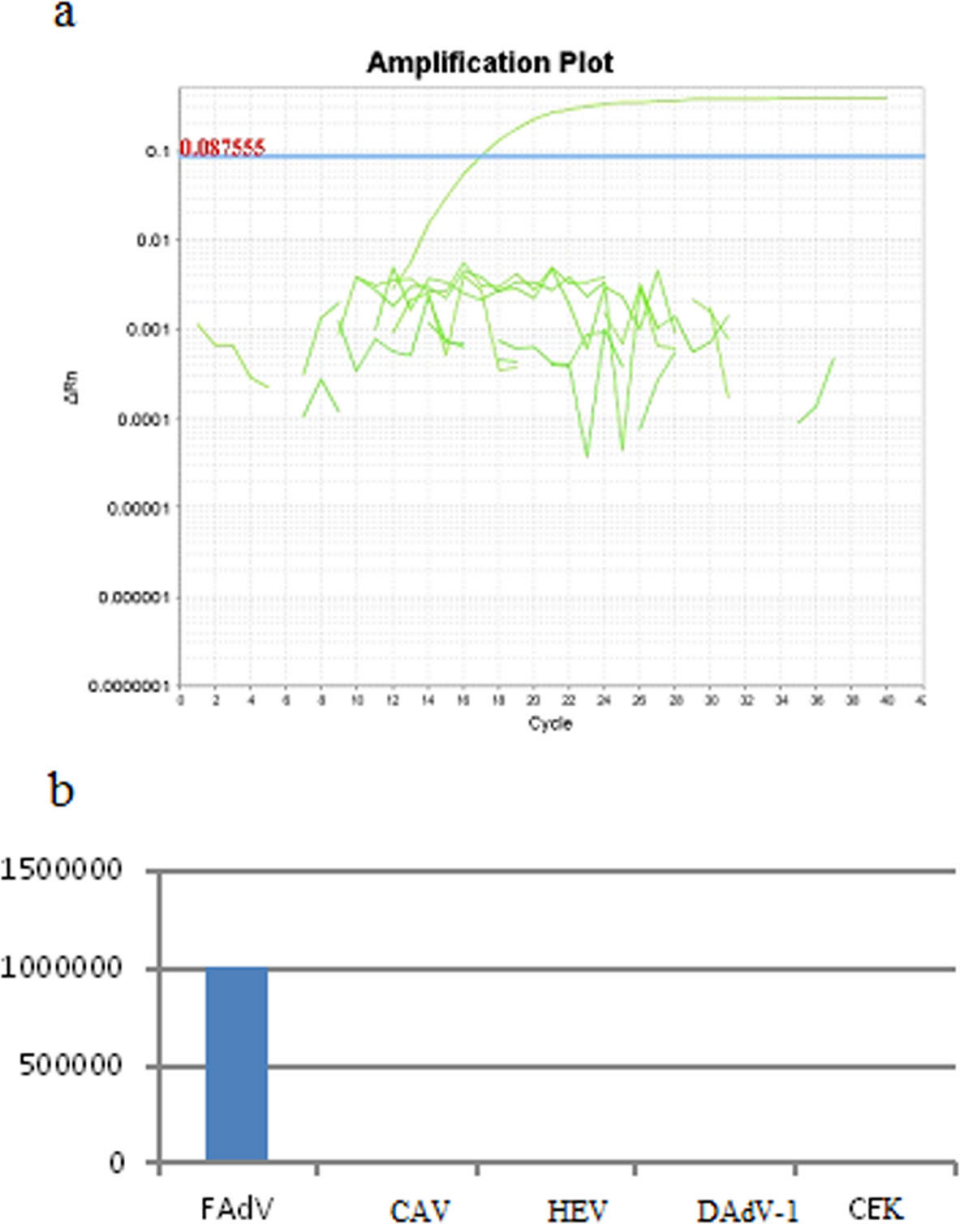
**a** Specificity of real-time PCR amplification melting temperature analysis of PCR products recorded in ABI 7500 Real-time PCR system (Applied Biosystems, Foster City, CA). Descriptions: NC - DNA extracted from non infected SPF chicken kidneys (CEKs). **b** Specificity NC - DNA extracted from non infected SPF chicken kidneys (CEKs). No real-time PCR curve was observed in the case of the negative control, nor for DNA obtained from other viruses: CAV, HEV, DAdV. Rel-time PCR curve was observed in the case of FAdV infection

**Fig. 3:**
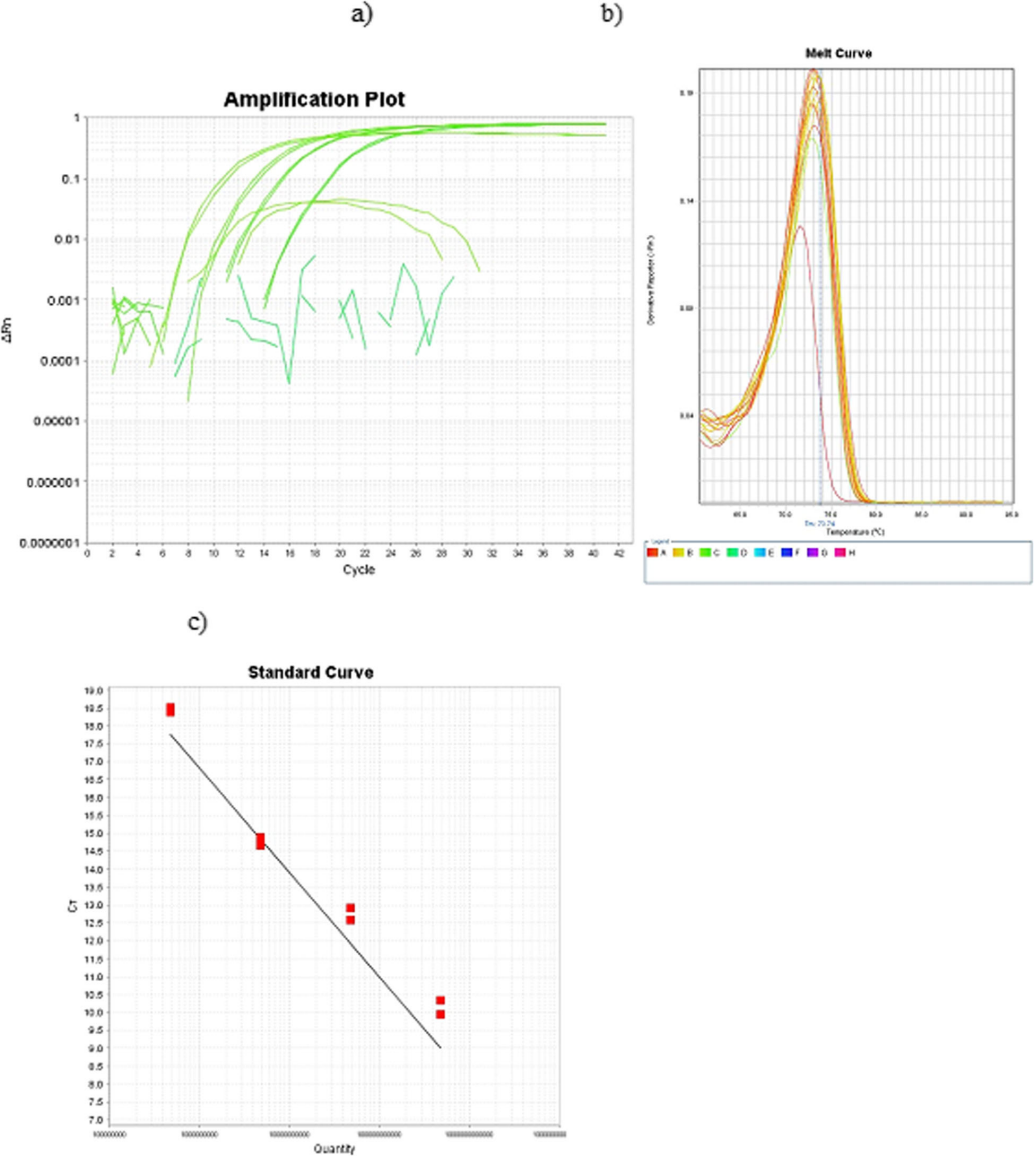
The sensitivity of real-time PCR. 1–4 appropriate dilutions (10^2^–10^5^ copy DNA/μl) adenovirus strain/species 2/D isolated from infected CEK by FAdV adenovirus strain. ΔRn is an increment of fluorescent signal during successive cycles. **a**, **b**, **c** The derivative reporter value is plotted as the y-axis while the temperature is plotted as the x-axis. The melting temperature peak is 73.74 °C (**b**). The standard curve derived during amplification has been obtained and presented in (**c**)

Tissue Culture Infectious Doses of field strains were determined in the 3rd passage as 3 log_10_TCID_50_/ml for FAdV-2/3/11/D respectively. In the next step of the study, total DNA was isolated and real-time PCR was applied. The optimal concentration of master-mix was set as 12.5 μl (u/μl). For total DNA, the optimal volume was chosen as 2.0 μl of DNA for FAdV with a concentration of 10 ng/μl, and a pair of primers with a concentration of 10 mM, and volumes of 1.5 μl each. Sequences of the designed primers and their location in region of the hexon gene for LAMP method are presented in Table 2, and the sequences of the designed primers and their location in region of the hexon gene are presented in Table 3. No real-time PCR curve was observed in the case of the negative control, nor for DNA obtained from other viruses (Fig. 2). The results of each reaction were determined by the calculation of the Ct values. In the next step, the sensitivity of real-time PCR was determined at the highest dilution of DNA in which a positive result was present with 10^2.0^ ng/μl DNA for FAdV-2/D, FAdV-3/D and FAdV-11/D respectively (Fig. 3). The primers were the most effective in 1.0 μl with a concentration of 10 ng/μl at 55 °C, amplicons of standard and field isolates correspond to the predicted curves compared to LAMP sensitivity which was determined according to Niczyporuk [21] as 10^2.0^ ng/μl of DNA.

**Table 2:** The positions of designed primer sequences within the hexon gene in the genome of FAdV-2/D KT862805 (ANJ02325), FAdV-3/D KT862807 (ANJ02399), and FAdV-11/D KC750784 (AGK29904) used in LAMP loop-mediated isothermal amplification

Gene	Name	Sequence	Genome Location	No. of bp.
hexon	F3 JSN	5’ACAACTACCTGTGGACCGT 3’	20,863–20,879	19
hexon	B3 JSN	5′ CGTTCGGGTTGGTTCACC 3’	21,041–21,059	18
hexon	LF JSN	5′ GGATTCTGACCCAGGTCCGT 3’	20,917–20,936	20
hexon	LB JSN	5′ CGAGAACACKTACGTSTACAT 3′	20,995–21,014	21
hexon	FIP(F1c + F2)JSN	5’TGCTGTGCGAGTTGTTGGTGTATTTTTCATGTACATGGGCGAACTG 3’	20,896–20,916/20,941–20,962	46
hexon	BIP(B1c + B2)JSN	5’ACTTCGAGTTGGACCCCATGGATTTTATGTCGAACACGCCGTAGA 3’	20,971–20,993/21,017–21,035	45

**Table 3:** The positions of designed primers and probe within hexon gene in the genome of FAdV-2/D KT862805(ANJ02325), FAdV-3/D KT862807 (ANJ02399) and FAdV-11/D KC750784 (AGK29904) used in Real - time PCR

Gene	Name	Sequence	Genome Location	Amplicon size
hexon	FAdV JSN-F	5’AATGTCACNACCGARAAGGC 3’	20,666–20,685	93
hexon	FAdV JSN-R	5’CBGCBTRCATGTACTGGTA 3’	20,759–20,739	93
hexon	JSN-FAdV probe	5′ AATCCCTACTCGAACACCCC 3’	20,739–20,760	–

NRB - degenerated bases

### LAMP visualization

**Fig. 4:**
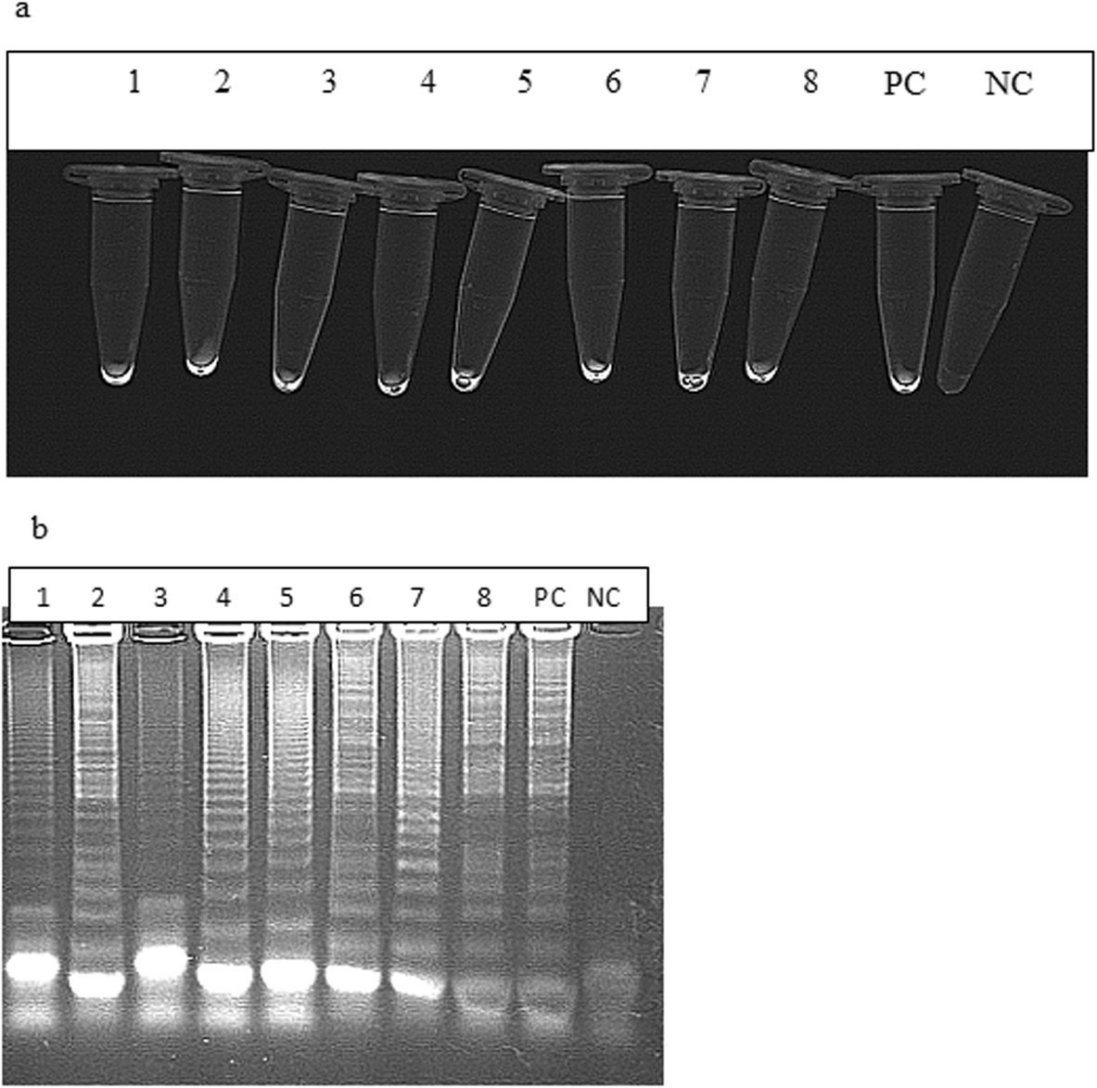
**a**, **b** The results of the visual LAMP detection method of FAdV DNA under UV light. The upper part shows **a** Observation of fluorescence of positive samples under UV light illumination. Descriptions: NC negative control - DNA template extracted from non infected CEKs, 1–8) appropriate adenovirus field strains detected in wild birds samples, 9) PC - positive control - DNA of FAdV-2/D strain of adenovirus product corresponding to – UV fluorescence. The lower part shows **b** gel electrophoresis of LAMP products with the presence of ladder-like bands.

LAMP showed a fluorescence in the positive control, and in 8 (2.52%) out of 317 DNA samples extracted from field specimens, marked as: A)-1/Msw1/16–2/D, B)-2/Msw2/16–2/D, C)-3Msw3/16–2/D, D)-4Wd1/16–2/D, E)5-Wd2/16–11/D, F)6-Wd3/16–11/D, G)7-owl1/16–11/D*,* H)8-wpi1/16–11/D, and have been presented in (Fig. 4a). Also specific bands were observed in electrophoresis only with the positive control and positive samples (Fig. 4b).

### Real-time PCR quantification

**Fig. 5:**
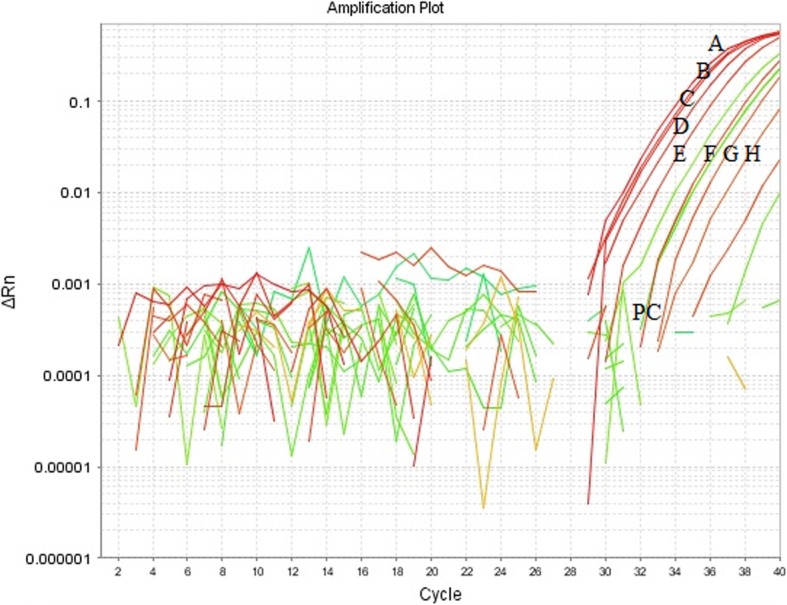
Results obtained by real-time PCR. Amplification plot of real-time PCR for the detection of adenovirus strains. ΔRn is an increment of the fluorescence signal during successive cycles specific for the positive control and samples 1–8, as described: NC-DNA extracted from non-infected SPF CEKs, PC - positive control, DNA extracted from positive control 2/D and 1–8 positive samples

**Fig. 6:**
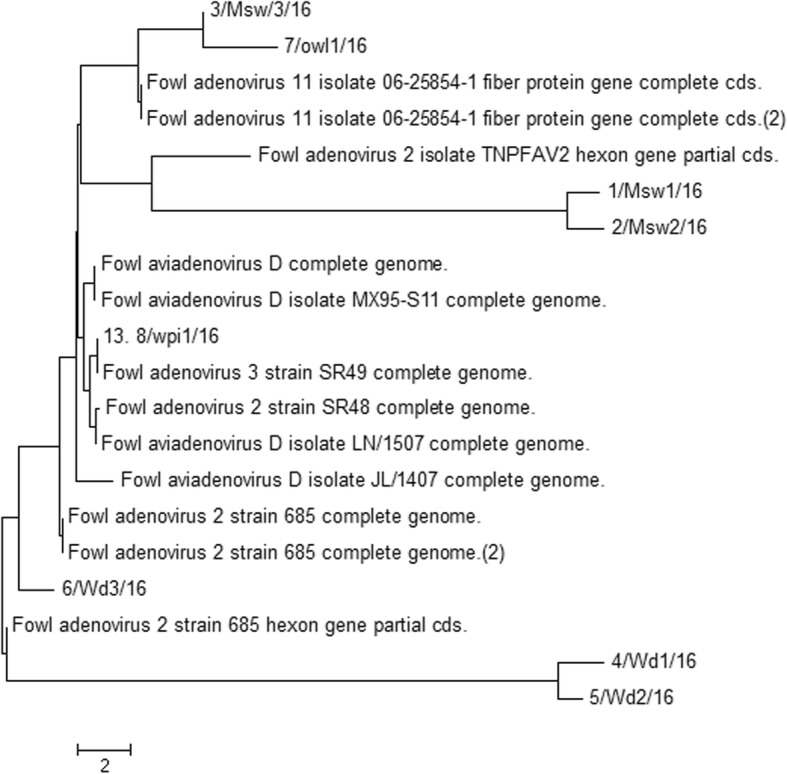
The phylogenetic tree was based on a region of 830 bp of the Loop L1 region of the hexon gene nucleotide sequence and was constructed using the maximum-likelihood method. The sequence of reference of adenovirus strains FAdV-2/3/11/D were used. Bootstrap percentages based on 1000 replicates are shown at the tree nodes. Evolutionary analysis was conducted using the MEGA7 software

In the next step of the study, real-time PCR was used for the analysis of field samples as a confirmation of the results obtained by LAMP. The real-time PCR showed the presence of fluorescence curves indicating the presence of adenovirus DNA in the positive control, and eight field samples designated as A-H, and positive control as PC (Fig. 5). A melting curve analysis creates a similar melting temperature Tm = 73.74 °C (Fig. 5) with *R*^2^ = 0.987, and efficiency = 98% for all amplified products which reacted. This peak was not observed in either the negative control nor negative samples. In eight among 317 DNA samples, the fluorescence curves were observed. The sequencing analysis of obtained amplicons indicated that detected virus strains were designated as types/species, FAdV-2/3/11/D (Fig. 6).

The samples detected by LAMP and quantified by real-time PCR were derived from different wild bird species which were as follows: three mute swans (*Cygnus olor*), three wild ducks (*Anas platyrhynchos*), one owl *(Strigiformes),* and one common wood pigeon (*Columba palumbus*).

## Discussion

Fowl adenoviruses are a very diverse group of agents, causing diseases in domestic and wild birds. Fowl adenoviruses are common in healthy birds as a ubiquitous pathogen, and can cause different diseases with mortalities ranging from 10% up to 90%, depending on the strain virulence observed in wild birds all over the world [4, 7]. The first adenovirus isolation from wild birds was described by McFerran [6]. Worldwide distribution and outbreaks of adenovirus infection in 40 wild birds species have been reported by many authors [12–14]. In the species Falcon adenovirus A belonging to the genus *Aviadenovirus* infection has been documented in merlins (*Falco columbarius*) [10], American kestrels (*Falco sparverius*), and Mauritius kestrels (*Falco punctatus*) [9], common buzzards (*Buteo buteo*) [12] red-bellied parrots (*Poicephalus rufiventris*) [7] and in tawny frogmouths (*Podagrus strigoides*) [13]. Strains pathogenic for poultry types FAdV-1/A and 4/C, are not always infectious for wild birds [2, 5]. Some adenoviruses were not virulent in their natural hosts, and may be more virulent when they cross the species barrier [2, 22] and, as in this study, where the FAdV-2/3/11/D has been isolated from wild birds. It is possible that Kites (*Milvus migrans*) can be infected after the consumption of sick chickens [8]. Different molecular diagnostic techniques have been used for adenovirus detection in wild bird samples [3, 8, 19, 21–27]. In some cases, detection was based on virus isolation, and serological investigation such as: agar gel immunodiffusion (AGID), enzyme-linked immunosorbent assays (ELISA), sero neutralisation (SN), and immunofluorescence essay (IFT) [28]. Because of the variability of the hexon gene in the nucleotide sequence, there is the possibility to differentiate several adenovirus types by real-time PCR and its modifications based on special primers and probes which have been designated for different adenovirus type/species [4, 17, 22]. Most of the studies were also conducted on serological methods, and avian adenovirus group-specific antibodies in serum samples of wild birds have been determined by AGID, SN, IFT, and ELISA [28]. Although in serologic studies it was impossible to discover which species can cause disease or if these birds were infected with avian or different adenovirus species [28]. The serologic survey reveals evidence of the natural exposure of free living common buzzards (*Buteo buteo*) in Germany, and the presence of adenovirus infection in Eurasian buzzards (*Buteo buteo*) [12].

Adenoviruses have been identified by electron microscopy in tissue samples from a Northern aplomado falcon (*Falco femoralis septentrionalis*) affected with inclusion body hepatitis and enteritis. Infection was confirmed in the brain sample of a goshawk (*Accipiter gentilis*) with neurological signs [29]. The presence of adenovirus strains were detected in a free-living tawny frogmouth (*Podargus strigoides*) in Australia with hemorrhagic enteritis symptoms described by Reece and Pass [13]. In the USA, Schelling [29] identified adenovirus infection in merlin (*F. columbarius*) with hepatitis disorders, and infection has also been detected by Forbes in 1997 in the UK in Mauritius kestrels (*F. punctatus*) with haemorrhagic enteritis, hepatitis, and sudden death.

A new pathogenic adenovirus species isolated from falcons has been recognised [11], which was very similar to fowl adenovirus types FAdV-1/A and 4/C. In our study, types/species 2/3/11/D have been detected. Primary lesions in affected falcons indicates inclusion body hepatitis, splenomegaly, and enteritis [11]. Adenoviral Gizzard Erosion (AGE) in broiler chickens, described as gizzard erosion and ulceration can lead to increasing mortality in a flocks however mostly lead to growth retardation caused mainly by type FAdV-1/A which was recognised over the last decade in Poland, and a specific method for detection, has been developed by Niczyporuk et al. [23] and Niczyporuk [21].

The results obtained by LAMP were fully confirmed by real-time PCR. Methods in birds was previously described by Xie [30] and in wild birds by Gunes [19]. The sensitivity allowed for a minimum detection limit of 9.4 viral genome copies. This was higher than the method obtained in this study with a sensitivity of 8 viral genome DNA copies/1 μl. The sensitivity was comparable with nested-PCR, and was 100 times more sensitive than conventional PCR. Other researchers also described PCRs for adenovirus detection in wild birds [7, 8, 15], and Heim [31] developed, optimised and performed real-time PCR for the detection of adenovirus strains in psittacine birds and humans, with concentrations ranging from 10 down to 8 copies per reaction. The sensitivity of real-time PCR using the SYBR Green chemistry PCR product is 6.6 and could not be detected in gel, however, the amplification plot and melting curves demonstrated specific fluorescence for adenoviruses.

The assay specific for targeting the fiber gene was not as sensitive as the one designated for the hexon gene. The fiber gene mRNA was presented in lower copy numbers than the hexon gene in mRNA [32]. Real-time PCR and LAMP for FAdV detection were 1000 times more sensitive than conventional PCR, duplex-PCR 10 TCID_50_ or triplex-PCR 10^2.0^TCID_50_ developed in a previous study by Niczyporuk et al. [23] and Niczyporuk [21]. Fowl adenovirus strains which had crossed the species barrier were present in approx. 2.52% of specimens of collected wild birds between 2015 and 2018.

## Conclusion

In conclusion, for the first time Xie et al. [30] developed LAMP method for the detection and identification of fowl adenovirus strains. To our knowledge this is the first study in which LAMP assay was applied for the detection of fowl adenoviruses in wild bird samples with the primers designed based on the basic sequences of the hexon gene which confirmed the specificity. During the study, in 8 (2.52%) out of 317 examined wild birds, the presence of fowl adenovirus strains was confirmed. The study on adenovirus strains in wild birds in Poland will be continued and the possible infection, and cross-species transmission between domestic and wild birds will be investigated. In addition the LAMP assay may be a useful alternative for the differential diagnosis of FAdVs. It is noteworthy that, although the quality of the analysed samples is characterised as low detectable viral load, the technique presented a good response in the detection. Comparing with real-time PCR which is cost consuming and the special equipment is needed in front of LAMP advantages.

## Methods

### Standard FAdV strains

FAdV-A-E standard strains were obtained as lyophylisates from (Charles River, US). The strains were propagated on CEK cultures, and the Tissue Culture Infection Dose (TCID_50_) virus titers were determined according to the Reed–Muench model [33].

### Other avian viruses

Chicken anaemia virus (CAV) 10^4.5^ TCID_50_/ml vaccine strain isolated from commercial vaccine, Siadenovirus, Hemorrhagic Enteritis Virus (HEV) reference strain obtained from ATCC with a virus titre of 10^4.5^ TCID_50_/ml and Atadenovirus, EDS (DAdV-1) with a virus titre of 10^4.5^ TCID_50_/ml reference strain from ATTC collection have been used.

### Sample collection

All birds selected for sampling were submitted for diagnosis were dead. Three hundred seventeen samples were collected from September 2015 to October 2018 from internal organs such as the spleen, liver, gizzards, intestine, and other pathologically changed tissues obtained from wild birds. The samples were derived and collected from different regions of Poland (Table 1). The samples were subjected to the LAMP method using the hexon gene Loop L1 region as a target. Every sample was examined separately by LAMP, and confirmed by real-time PCR. The samples were stored and archived at − 20 °C for the next stage of the study.

### CEK cultures

CEK cultures were prepared from 18 to 19 day old SPF chicken embryos (Lohman, Tierzucht, Cuxhaven, Germany) according to the standard procedure. The growth medium consisted of Eagle’s medium (MEM) with the addition of 10% of foetal bovine serum, and 0.1% of antibiotic mixture (Antibiotic–Antimycotic, Gibco, Scotland). The maintenance medium consisted of MEM with 0.1% of antibiotic mixture. A monolayer of CEK cultures was obtained after 18–24 h incubation at 37.5 °C in an atmosphere of 5% CO_2_.

### Isolation of adenovirus strains

After homogenisation, the tissue samples were submitted to three freeze-thaw cycles, filter sterilised through a Millipore filter with pores 450 nm in diameter, and 1 ml was used to inoculate nearly confluent CEK cells. The cell cultures were incubated for 5 days at 37.5 °C in a 5% CO_2_ atmosphere until the onset of the cytopathic effect. The samples which were found positive by LAMP and confirmed by real-time PCR were tested for virus isolation. When CPE was observed after three blind passages, the samples were considered to be positive.

### DNA extraction

Total DNA was extracted directly from prepared inoculates from the internal organs obtained from wild birds. The isolation was performed according to the commercial procedure using a DNA Mini Kit (Qiagen, Germany) from 200 μl of cell suspension. The extracted DNA templates were frozen and stored at − 20 °C until analysis.

### Loop-mediated-isothermal-amplification and primer selection

The nucleotide sequences of three pairs of primers used for the amplification of the FAdV hexon gene were previously designed, and all of the parameters were optimised by Niczyporuk [21]. LAMP was conducted as follows: final volume of 25 μl of mastermix containing 1x buffer Pol (50 mM Tris-HCl, pH 9.0, 50 mM NaCl, 5 mM MgCl_2_) (EurX), 1.6 M betaine (Sigma-Aldrich), 1.5 mM - dNTPs (EurX), 50 pM of FIP JSN and BIP JSN internal primers, 10 pM of F3 JSN and B3 JSN external primers, FIP (F1c + F2) JSN and BIP (B1c + B2) JSN loop primers, 8 U of polymerase *Bst* DNA (EurX), 2 μl DNA of 30 ng, and deionised water.

### LAMP specificity and sensitivity

The specificity, concerning the FAdV, CAV, HEV, and DAdV-1 viruses, and the sensitivity of the reference strains FAdV-2/D, 3/D and 11/D was published by Niczyporuk [21]. The electrophoresis of the amplicons was carried out in 2% agarose gel with 1 μg/ml of ethidium bromide, and was performed in a tris borate EDTA buffer, pH 8.2, with the use of Mini Sub-Cell (Biorad, USA) with 150 V and 80 mA for 50 min. The size of the amplification products were compared with a DNA Mass Ruler 1031 bp (Fermentas). The results were visualised and documented using transilluminator UV (Geno Smart VWR Germany).

### Real-time PCR confirmation

The sequences of nucleotide primers specific to the FAdV strains were designed based on the sequence of the Loop L1 HVR1–4 region of the hexon gene of reference strains FAdV-2/D KT862805 (ANJ02325), FAdV-3/D KT862807 (ANJ02399) and FAdV-11/D KC750784 (AGK29904) amplifying the 93 bp product, and were as follows: FAdV-JSN-F (sense primer): 5’AATGTCACNACCGARAAGGC3’ and FAdV-JSN-R (antisense primer) 5’CBGCBTRCATGTACTGGTA3’ and JSN-FAdV probe 5’AATCCCTACTCGAACACCCC3’ simultaneously for 2/3/11/D. Primers were designed in the Primer 3 programme according to the GeneBank database and synthesised in the commercial company Genomed in Warsaw. Cycle threshold (Ct) values from 23.07 to 24.51, indicating that there was no interference among the primers in the real-time PCR. Primers, were confirmed and used to evaluate the specificity and coverage. The real-time PCR have been performed by using Applied Biosystems 7500 Real-time PCR, and were used in a final volume of 25 μl. The mixture contained: 12.5 μl of PCR Master Mix, 1.0 μl of each primer FAdV-JSN-F and FAdV-JSN-R, 1.0 μl of probe, 2.0 μl of DNA, and 6.0 μl of deionised water. Nucleotides, Taq DNA Polymerase and buffer were included in the one-step RT- PCR kit (Qiagen). The designed protocol took 138 min. to obtain the results. The reaction conditions were as follows: 95 °C/15 min (initial denaturation), 94 °C/30 s (primer annealing), then 41 cycles of 55 °C/45 s (exact denaturation), 72 °C/1 min (signal acquisition). All reactions were carried out in duplicate with adequate melting curve analysis. As a positive control, total DNA extracted from the reference FAdV-2/D FAdV-3/D, and FAdV-11/D strains were used. As a negative control DNA obtained from non-infected CEK cells were used.

### Plasmid standards

pHexon plasmid was constructed by cloning a 93 bp fragment of the pHexon gene 2/D in pGEM-T Easy Vector (Promega, US) and Blue/White X-Gal/IPTG selection. The pHexon was then amplified in *E.coli* DH5α (Invitrogen, US) in a liquid LB medium with the addition of 100 μg/ml of ampicillin at 37 °C in a Max 4000Q apparatus (Barnstead/Lab-line). The extraction of plasmid DNA was isolated from 10 ml of liquid of an 18 h culture of *E.coli* DH5α cells using a Plasmid Maxi Kit (Qiagen, Germany).

### Quantification of FAdV

The quantity of the exact copy number of the hexon gene in the examined samples was calculated in Applied Biosystems software (Version 2.0.1). The quantification of the viral copy number was calculated according to a procedure previously described by Gunes et al. [19] and Steer et al. [20]. On the basis of serial tenfold dilutions of the pHexon, which produced from 10^2^ to 10^5^ DNA copies/1 μl, 4 point standard curves were prepared and used for the Hexon gene copy number calculation. The fluorescence curve indicated the amplification of specific fragments for the examined gene.

### Real-time PCR specificity and sensitivity

Whole cell DNA from CAV, HEV and DAdV-1 were used to determine the method specificity. Standard curves for plasmid detection showed a dynamic range and high correlation coefficient *R*^2^ > 0.99. Analysis with tenfold dilutions of whole cell DNA from the reference FAdV-2/D, 3/D and 11/D strains, which corresponds to DNA concentrations of between 10^2^ and 10^5^ DNA copies/1 μl were prepared. Taking Ct = 35 as the cut off value, the detection limit was 8 copies of virus genome (DNA) per reaction for 2/D, 3/D and 11/D respectively.

### Real-time PCR product analysis

The crossing point of the fluorescence curve and the threshold line were calculated automatically by the thermocycler software with a proportional baseline adjustment setting. The fluorescence level was measured after the end of each annealing step. The quantification data were determined through a comparison of the Ct values of the samples with the Ct of standards prepared with 10-fold dilutions of plasmid DNA. In order to detect the limit of the assay, a series of 10-fold dilutions from 10^2^ to 10^5^ of DNA were tested.

### Sequencing

The assembly obtained to prove positivity for FAdV nucleotide sequence alignments were sequenced using a GS FLX/Titanium sequencer (Roche, Switzerland) by GENOMED (Poland). Phylogenetic analyses were performed by comparing the obtained nucleotide sequences of field adenovirus strains with the sequences of adenovirus strains obtained from the GenBank (NCBI) database. A phylogenetic tree was generated by the Neighbor-Joining method with the use of the p-distance method (on 1000 bootstrapped data sets). Molecular analysis was performed using MEGA7, Geneious7, and BLAST computer software. On the basis of this analysis, the similarity between the examined adenovirus strains were determined [34–36].

## Data Availability

The datasets supporting the conclusions of this article are included within the article.
