# Correction to: Detection of fowl adenovirus D strains in wild birds in Poland by Loop-Mediated Isothermal Amplification (LAMP)

**DOI:** 10.1186/s12917-020-02491-4

**Published:** 2020-09-30

**Authors:** Jowita Samanta Niczyporuk, Wojciech Kozdruń, Hanna Czekaj, Natalia Styś-Fijoł, Karolina Piekarska

**Affiliations:** Department of Poultry Diseases, National Veterinary Research Institute, Partyzantow 57, 24-100 Pulawy, Poland

**Correction to: BMC Vet Res (2020) 16:58**

**https://doi.org/10.1186/s12917-020-2271-4**

The original article [1] contains errors in several sub-figures – namely: Fig. 1j, Fig. 3, and Fig. 4b. The corrected sub-figures can be viewed ahead. The original figure legends have not changed.

**Figure 1:**
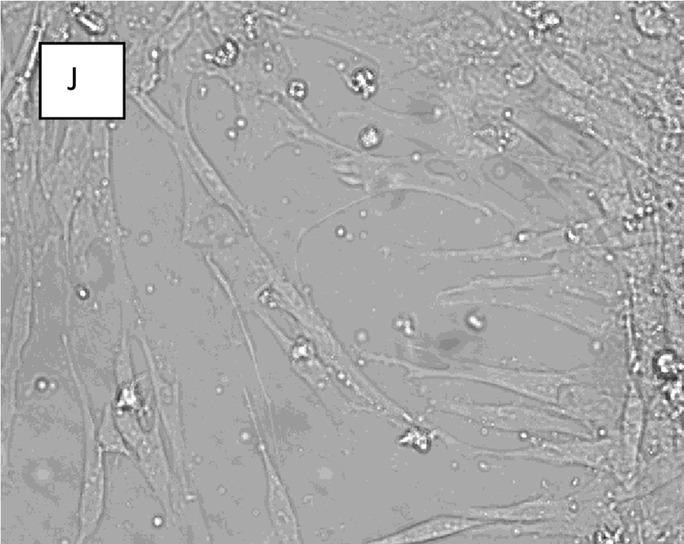
Fig. 1

**Figure 2:**
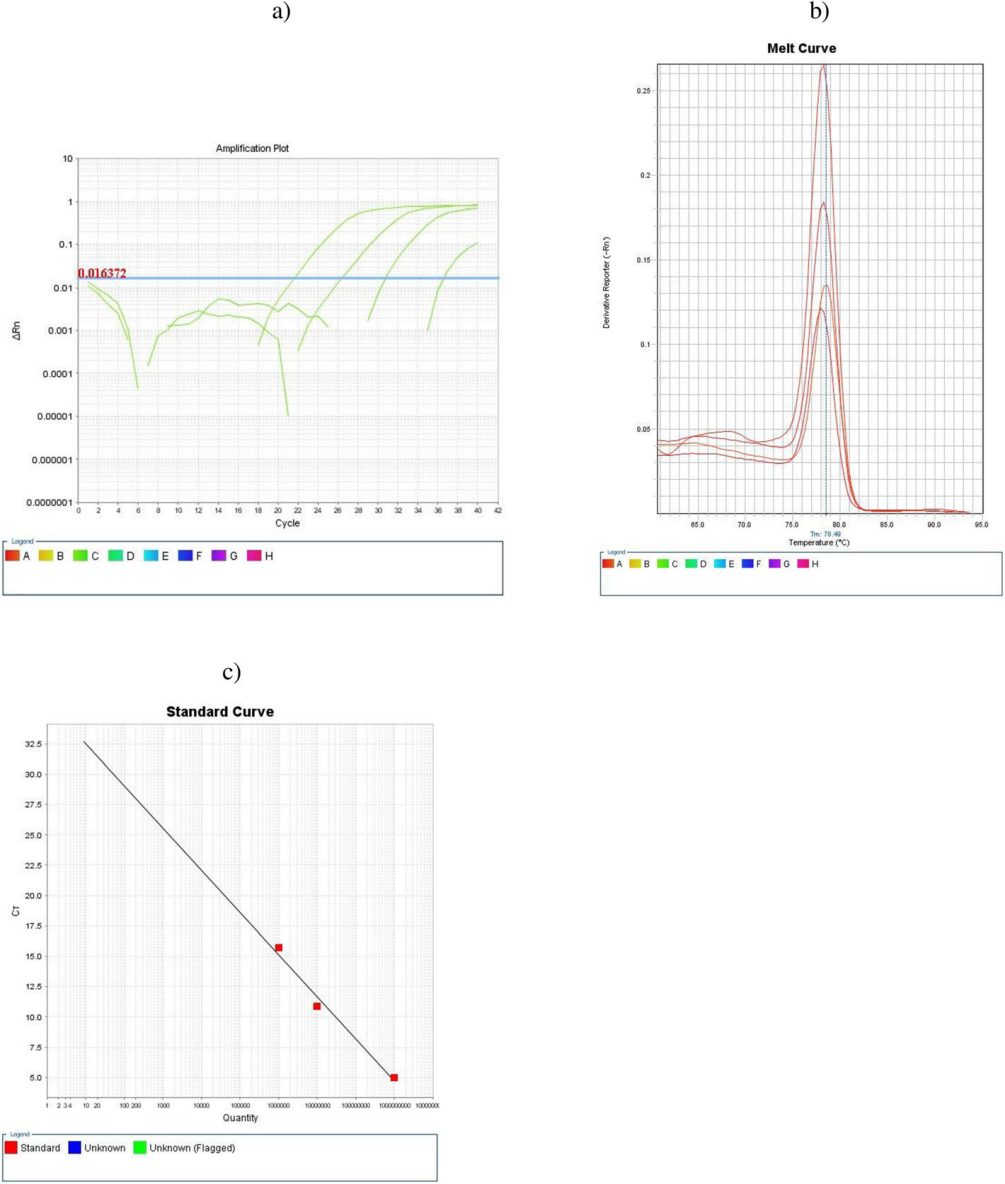
Fig. 3

**Figure 3:**
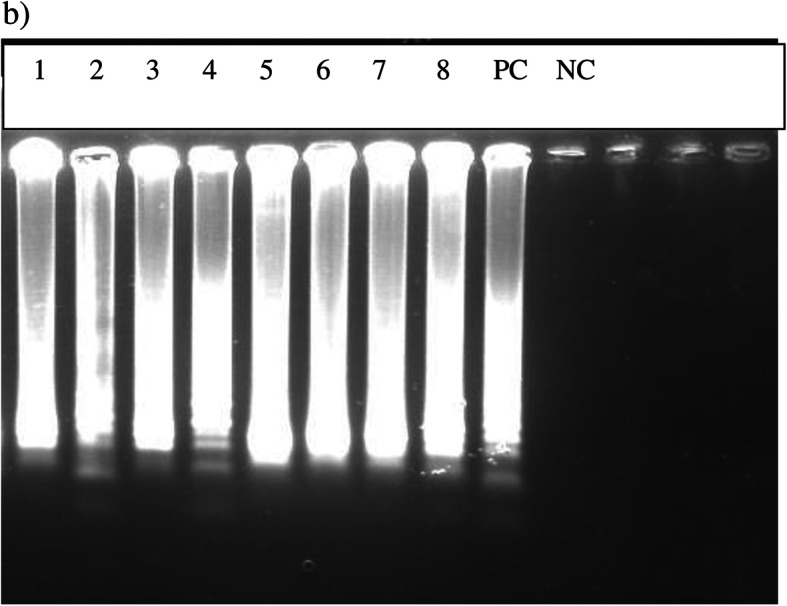
Fig. 4
